# Determinants of the Transmission Variation of Hand, Foot and Mouth Disease in China

**DOI:** 10.1371/journal.pone.0163789

**Published:** 2016-10-04

**Authors:** Jijun Zhao, Xinmin Li

**Affiliations:** 1 Institute of Complexity Science, Qingdao University, Qingdao, China; 2 School of Mathematics and Statistics, Qingdao University, Qingdao, China; Fudan University, CHINA

## Abstract

Severe outbreaks of hand, foot and mouth disease (HFMD) have occurred in China for decades. Our understanding of the HFMD transmission process and its determinants is still limited. In this paper, factors that affect the local variation of HFMD transmission process were studied. Three classes of factors, including meteorological, demographic and public health intervention factors, were carefully selected and their effects on HFMD transmission were investigated with Pearson’s correlation coefficient and multiple linear regression models. The determining factors for the variation of HFMD transmission were different for the southeastern and the northwestern regions of China. In the northwest, fadeouts occurred yearly, and the average age at infection and the fadeout were negatively correlated with the population density. In the southeast, HFMD transmission was governed by the combined effects of the birth rate, the relative humidity and the interaction of the Health System Performance and the log of the population density. When the Health System Performance was low, HFMD transmission increased with the population density, but when the Health System Performance was high, the better health performance counteracted the transmission increase due to the higher population density.

## Introduction

Hand foot and mouth disease (HFMD) is a childhood infectious disease that is principally caused by enterovirus 71 (EV71) and coxsackievirus A16 (Cox A16). HFMD has circulated in East and Pacific Asia for decades [1,2]. In China, HFMD shows characteristic spatial incidence patterns [3,4]. Spatially, the annual peak timing of incidence seasonality varied from April in the southern region to July in the northernmost region [3]; additionally, the number of HFMD cases reported varied greatly from the south to the north and from the east to west [5,6]. Research effort about HFMD spatio-temporal patterns in China has mainly focused on spatial clustering in a locale [7,8], effects of meteorological factors on cases seasonality [9,10]. There has been only a few studies on the factors that determine the spatial incidence patterns [3–6,11,12], and these investigations have focused on the association of the climatic or social factors with the nationwide variation in the number of reported cases.

Enteroviruses outside of the host can be highly resistant to ambient environmental conditions [13]. Moreover, no experiments have been performed that directly measured the variation of the survival of the HFMD virus in the air, on fomites and in the food with respect to ambient humidity or temperature. Nevertheless, the variation in the HFMD occurrence or the amplitude of the seasonality peaks was associated with meteorological factors including rainfall [3–6,11], wind speed [4,6], relative humidity [4,11], temperature [4–6,11], air pressure [3,5] and sunshine [3]. Some meteorological factors were significantly associated with the nationwide variation in HFMD occurrence in some studies, but not in others. Geographical variation in HFMD reported cases was also associated with socio-economic factors, such as the population density, the proportion of the student population, and the number of enterprises above a designated size [4,6,11,12]. Disagreements exist about the type or combination of factors that are most responsible for the variation of HFMD occurrence in China, which shows great demographic, social and economic differences among provinces. For example, the child population density, the number of enterprises above a designated size [6], the tertiary industry were reported to be dominant in the determinant factors in studies of [11], [6] and [12] respectively. The disagreements maybe due to the selection of potential factors. Besides, a bias may exist in the association of the HFMD spatial patterns with possible determinants, due to the large variation in reporting fraction (the proportion of infections that are reported to the monitor system) across the country [14] that has not been considered in these studies. The large variation in reporting fraction was reported for some childhood infectious in Europe and Mexico [15,16], and reporting fraction was usually considered in models of these diseases.

Severe outbreaks of HFMD had occurred in the southeast provinces of China, and many fewer cases were reported in the northwest provinces than in the southeast provinces. There were fadeouts (no cases or only very few were reported) occurred in the northwest provinces. The drivers of the HFMD transmission in the northwest provinces and southeast provinces might not be similar. However, different drivers of HFMD transmission have not been studied up to now.

The disease incidence patterns are “observations” of a transmission system and are determined by the transmission process of a disease. Hence once we understand the transmission characteristics of a disease we can understand its incidence patterns. In the previous studies about the nationwide variation of HFMD occurrence, possible determining factors were directly associated with observed HFMD cases or dependent variables calculated from reported cases. Many insights have been found; however, our understanding of the HFMD transmission process is still limited. Spatial incidence patterns could be caused by heterogeneity in local transmission characteristics of the disease. Understanding the factors that determine the transmission characteristics can help us to understand the drivers of the HFMD transmission process, explain its spatial incidence patterns, and is critical in the planning of effective control measures.

National differences in transmission characteristics such as the average age at infection reflect local variations in the dynamic process of HFMD transmission. The average age at infection is a transmission characteristic that can describe the transmission level and the transmission process. The average age at infection of HFMD was reported to vary across the country [14]. National variation of the average age at infection explained one of the spatial pattern, the variation of the annual peak timing of HFMD seasonality. Regions that showed a lower average age at infection had an earlier annual peak timing in the spring [14]. The average age at infection was inversely related to the individual risk of infection and also to the transmission rate [17]. Hence, if a region had a higher transmission rate or a lower average age of HFMD infection, an earlier annual peak timing of HFMD seasonality in the region would be observed.

Factors that determine the variation of the average age at infection have been studied for other childhood diseases. Particularly, a shift in the average age at infection from the pre-vaccination era to the post-vaccination era has been predicted [18] and has been well documented as a general phenomenon for rubella, measles, pertussis, etc. [16,19,20]. The decrease in the incidence as a consequence of vaccination was expected to reduce the force of infection and increase the average age at infection [18,20,21]. Spatial variation in the average age at infection of measles after vaccination had been reported in different countries, and the variation was shown to be due to both the vaccination coverage and the birth rate [22]. Spatial variation of the average age at infection before vaccination was observed for measles in different regions in Italy [23], and the reasons behind it were episodic outbreaks (i.e., highly irregular multi-year outbreaks). The average age at infection of HFMD in China varied spatially, especially between the northwestern and the southeastern regions [14]. The reasons for such variation of the average age at infection of HFMD have not been investigated.

Theoretically, the amplitude of a transmission characteristic such as the average age at infection is determined by many factors. Based on an environmental infection transmission system (EITS) model [24,25], the amount of the virus that accumulates in the environment, the pickup rate of the pathogen from the environment, the population density and the number of susceptible individuals determine the transmission of a disease transmitted through the environment. The amount of the virus that accumulates in the environment can be affected by meteorological factors and population density, and sometimes the weather can also change human behaviour to strengthen or weaken the effect of population density. Health care interventions, such as routinely cleaning fomites, decreasing the pathogens in the air, decreasing the contamination of food and water, and hand washing, could decrease transmission by decreasing the amount of the virus in the environment or decreasing pickup rate. Birth rate and vaccination change the number of susceptible individuals. The birth of new babies refreshes the susceptible population, and vaccination removes partial susceptible population to the recovered. Hence, theoretically, population density, number of susceptibles (changed by birth rate and vaccination), climatic and health care intervention factors should determine the geographical variation of transmission of a disease. For childhood diseases such as rubella, measles and pertussis, vaccination and birth rate dominant the factors that determine the variation of transmission as describe in the previous paragraph. Although population density and public health intervention have not been studied specifically for their effects on transmission variation, high population density and poor sanitation that can be highly improved by health care intervention, were reported being highly favourable for the transmission of polio [26,27]. Climatic factors can cause transmission seasonality due to the fact that they seasonally change population density in a region [28], however their effects on the geographical variation of transmission of a disease has not been reported so far.

In this paper, we took the average age at infection as an index of the transmission of HFMD and studied the determining factors of the variation in the average age at infection of HFMD in Chinese provinces. First, we selected three classes of some inferred factors in the regions studied: meteorological, demographic and public health intervention factors. Then we evaluated the effects of these factors on the average age at infection using a correlation analysis and multiple linear regression models, and we discovered some significant factors that caused local variations in HFMD transmission. Finally, we studied the different drivers of transmission in northwest provinces and in southeast provinces in China.

## Methods

### Data

We obtained the number of reported cases of HFMD from the Chinese CDC [29]. The dataset covered the age-stratified HFMD cases in mainland China from May 2008 to December 2011 for 31 provinces and municipalities. Hong Kong, Macau and Taiwan were not included. We used the term “provinces” for municipalities in this paper for simplification. The average age at infection in the provinces from May 2008 to December 2011 was calculated in our previous study and was based on the above data set [14] using an ODE model. Since the data did not distinguish the virus type, we did not account for EV71 and A16 separately, and we assumed that their transmission characteristics were similar. This assumption is reasonable, because the age distributions of EV71 and A16 during 2008–2012 were quite similar [3].

The meteorological data for each province (except Tibet) from 2008 to 2011 were obtained from the China Meteorological Data Sharing Service System [30]. The data included the mean temperature, mean relative humidity, mean rainfall, and the mean hours of sunshine.

Demographic data, including the birth rate, the province population, and the GRP, were obtained from National Bureau of Statistics of China [31]. The population density was calculated from the province population divided by the province land area. The data that quantify the public health intervention level in provinces was from the study of Liu et al. [32] and was calculated from their published data on the performance indicators of health system coverage.

We did not analyse data in finer scale, such as county level, because there were high correlations between subpopulations in county level such that subpopulations act like a well-mixed population. Province level is a sufficiently large scale to observe necessary dynamics. Similarly, state level scale was used in an analysis of polio in United States [33].

### Selection of factors

As introduced in the Introduction section, theoretically, meteorological, demographic (i.e., the population density and the birth rate) and public health intervention factors probably determine the HFMD transmission rate and hence, the average age at HFMD infection. It is strongly possible that the variability in demographic factors interacts with climatic seasonal stimuli and public health intervention to govern disease epidemics. In the following, we will select possible determining factors for the HFMD transmission.

1) Meteorological factors

The mean temperature, maximum temperature, minimum temperature, dew point, atmospheric pressure and evaporation were highly correlated with each other [34], and the total of bright sunshine and solar radiation were also highly inter-related [34]. Of these factors, considering the availability of the data, we included only the mean temperature and the mean hours of sunshine in our model, and excluded other highly correlated factors. Rain and relative humidity were also included in the model. We did not consider wind speed because virus transmission via airflow is mainly indoor or in-building transmission [35].

2) Factors that would change susceptible individual numbers

The population density and the population density of children of certain ages were reported to be associated with the occurrence of HFMD or with case ratios for children of certain ages [4,6,11]. These two factors are highly correlated. Since population size were usually considered in the analysis of other childhood infections, we only included population density in our model.

Birth rates in Chinese provinces were relatively stable during 2008 to 2011, however the average birth rates in this time period in provinces varied from 6.86‰ (in Jilin) to 15.76‰ (in Xinjiang). The birth rate was included in our model. Because there had been no vaccination for HFMD during the time period of our data, vaccination effect will not be considered in this paper.

3) Public health intervention factors

To quantify public health intervention level in the provinces, we used an index called the Health System Performance, which was calculated from a comprehensive set of health system performance indicators for Chinese provinces [32]. The performance indicators summarized the overall pattern of public health service delivery at the provincial level. To measure the effective coverage of health intervention, Liu et al. selected representative interventions that were relevant for China’s major health problems and could be measured in all of the provinces [32]. The Health System Performance in our study was calculated as an average of 11 performance indicators from Table 2 of Liu et al. In Liu et al., the 11 performance indicators were grouped into four major types, including “curative interventions to treat different diseases”, “preventive interventions”, “behavioural interventions” and “intersector public health interventions” for example, regarding drinking water [32]. The geographical distribution of the performance indicators was noteworthy–the southeast provinces showed better performance indicators than the western provinces [32].

We assumed that the implementation of public health control in a province could be facilitated by a good economic development. A more economically developed province might have more resources to allocate to disease control and its population might be more willing to implement intervention strategies. We used the per capita GRP (gross regional product, which is a measure of a region’s economic performance) as an indicator of the economic development of a province, and we designated the per capita GRP and the Health System Performance as the public health intervention factors.

We examined a total of eight factors in our model: the mean temperature, the mean hours of sunshine, the rainfall, the relative humidity, the population density, the birth rate, the Health System Performance and the per capita GRP.

### The correlation analysis and the regression model

We examined the association between the average age at infection and each factor and the correlation among the factors using Pearson’s correlation coefficient. To analyse the effect of the factors on HFMD activity, we entered three classes of factors (i.e., demographic factors, public health intervention factors, and meteorological factors) into a multiple linear regression model. Because excessive correlations were present among the four meteorological factors and high correlations were present among the demographic and health intervention factors, collinearity could prevent the identification of explanatory variables in our model. One approach to cope with this problem is to identify collinearity among factors by using variance inflation factors (VIF) and then remove individual factors that have high VIF values [36]. A backward approach was used until all factor VIF values were below a given threshold. For this study, we chose a VIF threshold value of 5. After all factors with a higher VIF value were removed from the model, we used an analysis of variance, subtracting factors in a stepwise manner until the best fitting model was found. In this way, we identified the factors that caused significant national variance of the average age at infection. The R software, version 3.1.0 was used for the correlation analysis and the regression models.

To examine different drivers of the average age at HFMD infection in the northwestern and southeastern regions, we divided the 31 provinces into two groups: nine provinces in the northwest and 22 provinces in the southeast (see S1 File). Besides analysing the overall data, we analysed two regions separately for the determining factors of the average age at infection. Because we did not have meteorological data for Tibet, we used data for only 30 provinces in the data association and linear regression model for the entire country and we used data for only eight provinces in the data analysis for the northwestern region.

HFMD fadeouts occurred in the northwestern regional provinces. We associated the fadeout with each of the three factor classes with Pearson’s correlation coefficient.

## Results

Since the average age at infection is inversely related to the transmission rate, we used it as a transmissibility index for HFMD to investigate the determinants of HFMD transmission.

### Determinants of HFMD transmission in the northwestern region

We only had meteorological data from eight provinces in the northwestern region. Even though we used six factors, a multiple linear regression model for the northwestern region could not be found. Hence we examined the Pearson’s correlation coefficients for each factor with the average age at infection in the northwestern region (Table 1). Regard to the Pearson’s correlation coefficients for each factor with the average age at infection for the entire country and the southeastern region, readers can refer Table A in S2 File.

**Table 1:** Correlation coefficient between each factor and the average age at infection and coefficients of the fadeout with factors for the northwestern region.

Factors	Correlation with *A*^1^ Coefficient (*p*-value)	Correlation with fadeout Coefficient (*p*-value)
Mean temperature	-0.2333 (0.5782)	-0.4665 (0.2439)
Relative humidity	-0.1651 (0.6961)	-0.2389 (0.5689)
Rainfall	-0.2344 (0.5763)	-0.2880 (0.4892)
Sunshine	0.1783 (0.6727)	-0.2965 (0.4758)
Log (population density)	-0.6803 (0.0437)*	-0.8638 (0.0027)*
Health System Performance	-0.2032 (0.6000)	-0.4581 (0.2150)
Birth rate	0.1626 (0.6761)	0.6997 (0.0359)*
Per capita GRP	-0.1202 (0.7580)	-0.5841 (0.0987)

^1^ *A* is the average age of infection

For the eight provinces in the northwestern region, the average age at infection was negatively associated with the log of the population density, but not significantly associated with any of the other factors.

**Fig 1:**
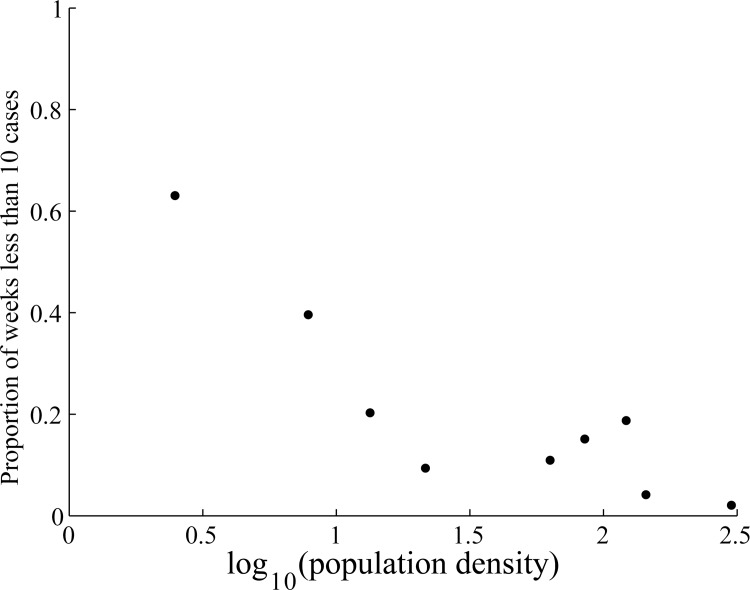
Proportion of weeks with less than 10 cases vs. the log of the population density in nine provinces in the northwestern region. Note, Tibet is included in this figure.

In the winter in the northwestern region, no cases or only very few were reported during some weeks. The proportion of stochastic fadeout (weeks with less than 10 cases in a province) was negatively associated with the log of the population density (p-value <0.005), positively associated with the birth rate, and was not associated with any other factor (the 3^rd^ column on Table 1). During 2008 to 2011, the birth rate in the northwestern region was negatively associated with the population density with a correlation coefficient of -0.65 (p-value <0.05). When considering only the relationship between the fadeout and the population density, the proportion of weeks with stochastic fadeout decreased with the log of the population density (Fig 1), similarly to findings for other diseases.

In the northwestern region, HFMD had a fadeout during most years, meaning that HFMD was frequently re-introduced into the region. After introduction into the population, the transmission in a province was higher if the population density was relatively high.

### Determinants of HFMD transmission in the southeastern region and the entire country

We first checked the correlations between factors. In the entire country and in the southeastern region, the four climatic factors were highly correlated with each other with Pearson correlation coefficients ranging from 0.711 to 0.996. The log of the population density, the birth rate and the per capita GRP were correlated with each other with Pearson correlation coefficients ranging from 0.56 to 0.89. Please refer to Tables B to D in S3 File for the specific correlation coefficients.

Because the factors were highly correlated for the entire country and the southeastern region, we used multiple linear regression models for our analysis, as guided by the VIF selection. Tables E and F in S4 File explains the VIF values calculated for each step.

Nationally, the average age at infection was associated with the relative humidity, the log of the population density, the birth rate and the interaction of the Health System Performance and the log of the population density (see the 2nd to 5th columns of Table 2). The *p*-value of the coefficient of the Health System Performance was high (0.9); however, we kept it in the model because an interaction was present between this factor and the log of the population density.

**Table 2:** Regression models for the country and the southeastern region.

	Country				Southeastern region			
Factors	Estimate	Std. Error	t value	*p*-value	Estimate	Std. Error	t value	*p*-value
(Intercept)	6.734	0.513	13.122	2e-12	4.520	0.693	7.069	2e-06
Relative humidity	-0.020	0.006	-3.506	0.0018	-0.011	0.006	-2.011	0.0614
log (PopDen) ^1^	-0.189	0.045	-4.21	0.0003	0.049	0.064	0.771	0.4519
Health System Performance	-0.001	0.008	0.120	0.9057	0.000	0.0067	-0.053	0.958
Birth rate	-0.107	0.021	-5.007	4e-05	-0.087	0.027	-3.170	0.0059
Log (PopDen):Health^2^	0.015	0.0038	3.944	0.0006	0.033	0.008	4.085	0.0009

^1^ Log (PopDen) is the log of the population density

^2^ Log (PopDen):Health is the interaction of the log of the population density and the Health System Performance.

In the southeastern region, the average age at infection was associated with the relative humidity, the birth rate, and the interaction of the log of the population density and the Health System Performance (see the 6th to 9th columns of Table 2). The *p*-values of the coefficients for the Health System Performance and the log of the population density were high; however, we kept them in the model because an interaction was present between them.

The model variances that could be explained by variables are listed in Table 3. The relative humidity could explain 39.2% of the variance of the average age at infection in the country, and 32.9% of the variance in the southeastern region. The demographic factors and the interaction together could explain more than 40% of the variance of the average age at infection in the country and in the southeastern region. The multiple linear regression models explained a high percentage of the variance in the average age at infection in the country and in the southeastern region.

**Table 3:** ANOVA table for regression models for the entire country and the southeastern region.

	Country					Southeastern region				
Factors	df	Sum sq	F value	*p*-value	Variance explained	df	Sum sq	F value	*p*-value	Variance explained
Relative Humidity	1	2.552	57.307	8e-08	39.2%	1	0.744	27.413	8e-05	32.9%
log (PopDen)^1^	1	1.202	27.000	3e-05	18.5%	1	0.239	8.812	0.0091	10.6%
Health System Performance	1	0.179	4.018	0.0564	2.8%	1	0.00591	0.217	0.6473	0.3%
Birth rate	1	0.812	18.224	0.0003	12.5%	1	0.389	14.306	0.0016	17.2%
Log(PopDen): Health^2^	1	0.693	15.552	0.0006	10.6%	1	0.453	16.689	0.0009	20.0%
Residuals	24	1.069				16				
Multiple *R*^2^	0.836					0.808				

^1^ Log (PopDen) is the log of the population density

^2^ Log (PopDen):Health is the interaction of the log of the population density and the Health System Performance.

Determinants for HFMD transmission in the southeastern region and in the entire country were similar. The transmission was affected by the combined effects of the relative humidity, the birth rate and the interaction of the Health System Performance and the population density.

**Fig 2:**
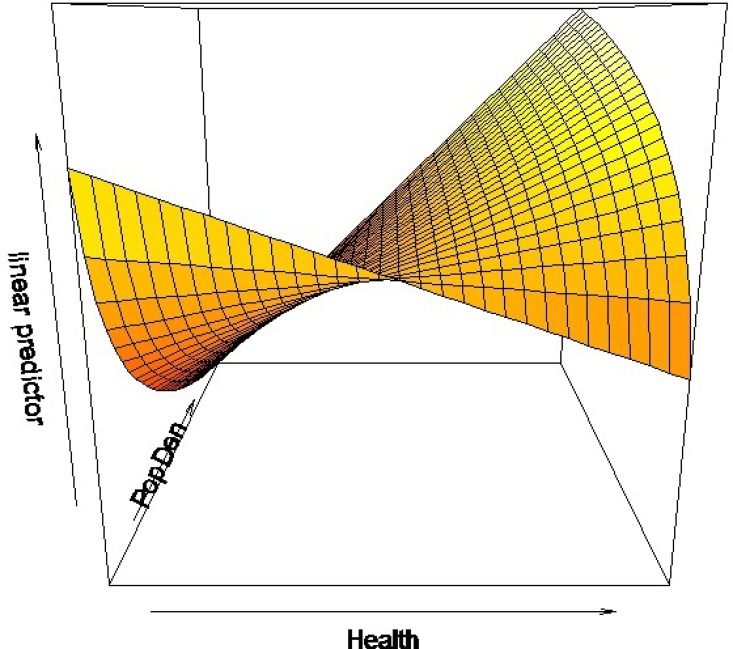
The linear predictor of the average age at infection as a function of the population density and the Health System Performance in the southeastern region.

**Fig 3:**
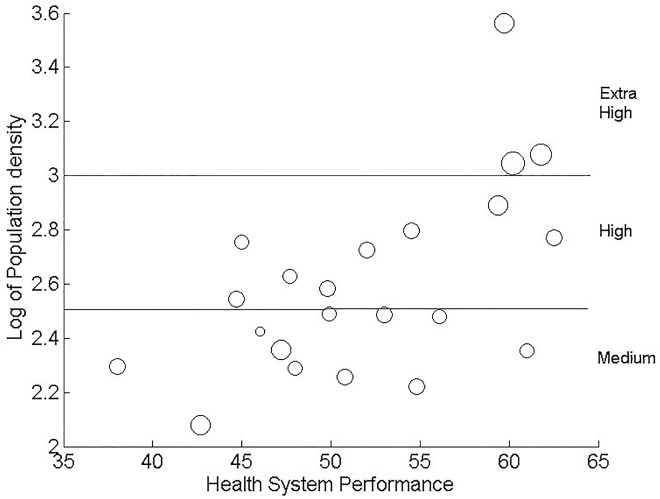
The observed average age at infection. The circle sizes are proportional to the values of the average age at infection.

In general, the population density should be negatively related to the average age of infection (i.e., positively related to the transmission rate) as shown in the result for the model of the entire country. However, the estimate of the log of the population density in the model of the southeastern region was positive. In order to understand the effect of the population density and its interaction with the Health System Performance, we plotted the predictor of the average age at infection with respect to the population density and the Health System Performance (Fig 2). To facilitate expression, we divided the population density into three levels: extra high, high, and medium (Fig 3). The provinces that had an extra high population density were Shanghai, Beijing and Tianjin. Note that the population densities of most of the provinces in the northwestern region that are not shown in Figs 2 and 3 were lower than the population densities of the 22 provinces in the southeastern region, and we assigned them to a low level. There were no provinces that had an extra high population density with a low Health System Performance (upper left part in Fig 3).

Provinces that had a low Health System Performance had an average age at infection that was negatively related to population density (Fig 2). This result was similar to the northwestern region in which the provincial population densities were relatively lower than those in the southeastern region. In general, a high population density increased disease transmission, reducing the average age at infection. However, in the southeastern region in provinces that had a higher level of Health System Performance, the effects of the Health System Performance compensated for the decrease in the average age at infection as a result of the population density. Especially, the effect of the Health System Performance is even higher at a higher population density. Therefore, provinces with a high Health System Performance and a high or extra high population density had a higher average age at infection. We found that the Chinese provinces that had the highest value of the product of population density and Health System Performance were mostly economically developed and were along the east coast, including Guangdong, Zhejiang, Shanghai, Jiangsu, Shandong, Tianjin and Beijing.

Provinces that had higher transmission potential or a lower average age at infection, for example, HeNan, Anhui, Hubei, Hunan, Fujian, etc., had a high to medium population density and a relatively lower Health System Performance.

## Discussion

We studied the response of HFMD transmission to meteorological, demographic and public health intervention factors using the average age at infection calculated from age-specific incidence data at the province level. We identified the response patterns of HFMD infection to these factors and discovered which factors affected transmission in the northwestern and the southeastern regions of China.

The results of our multiple linear regression model showed that the nationwide transmission of HFMD was due to the combined effects of relative humidity, the log of the population density, the birth rate and the interaction of the log of the population density and the Health System Performance. Until now, most studies that investigated the seasonality and the nationwide variation of HFMD occurrence have focused on the effects of meteorological factors. Meteorological factors are important because the effects of the ambient environment on the survival of the virus have long been considered to be related to the seasonality of many diseases. Our results also showed that relative humidity was a significant factor that affected HFMD transmission in the country. However, besides the meteorological factors, the importance of other factors is evident. In the southeastern region of China in which severe HFMD outbreaks occurred, the relative humidity was only weakly related to the number of HFMD case reports, while the birth rate and the interaction of the public health performance and the population density were significant for HFMD transmission. The birth rate and the public health performance had not been investigated in previous studies.

Regarding the interactions of factors on HFMD transmission in the southeastern region, when the Health System Performance was relatively low, the average age at infection was negatively related to the population density. In this situation, a province with a relatively higher population density would have a lower average age at infection (or higher transmission potential). For provinces that had a high Health System Performance, the better health performance counteracted the increase in transmission due to the higher population density. Hence, even when the population density is high, high performance in public health intervention still can remarkably reduce HFMD transmission. In China, the majority of provinces with high population densities are along the east coast, and these provinces are also more economically developed and have a higher Health System Performance than the other provinces.

The mechanism of the variation of the average age at HFMD infection in the northwestern region is different than that for the southeastern region. In the northwestern region, fadeouts occurred almost every year, mainly in December or January. HFMD cases began in February after the Chinese spring festivals. The occurrence of HFMD in this region might due to the migration of people between the northwestern and the southeastern regions, especially during spring festival week in January or February, depending on the Chinese lunar calendar. Once it was introduced into the population, virus transmission and fadeout then depended on the population density. The average age at infection or the transmission rate was not associated with climatic factors and the public health performance in the northwestern region.

We made some assumptions regarding the use of the selected factors. The Health System Performance values for the provinces were calculated from the 2005 to 2008 data [32]. We did not update the Health System Performance due a limitation in the source data, and hence we assumed that the relative strength of the provincial Health System Performance from 2008 to 2011 was similar to 2005 to 2008. An updated performance index might have given more accurate model results. Still, our results showed a significant effect of the interaction of the Health System Performance and the population density. A province that had a higher Health System Performance should have better intervention for control of HFMD. A good health system in a province also meant that the province had a good water treatment, sewage systems etc. and likely had good community hygiene overall. Regarding economic development factors, several different indexes, such as the number of enterprises above a designed size, tertiary industry, GDP, and first industry, were assessed in a study for their relationship with HFMD occurrence [12]. In our study, we only considered that economic development might help facilitate the implementation of public health intervention and we supposed that the per capita GNP could be used as the index for provincial economic development. Some factors, such as visiting an outpatient clinic for other reasons, or neighbourhood children congregating in a playground, have important roles in transmission [37]. Because factors such as these reflect population aggregation, we did not specifically consider their effects.

Disagreement of the results of previous studies regarding the driving factors of the variation in the number of cases might be because reporting fractions were not considered in those studies. The reporting fraction of HFMD ranged from 2% to 25% in Chinese provinces [14]. The use of reported cases data without considering the variation in the reporting fraction may cause a bias in the effects of associated factors on the HFMD transmission and may not reveal the true relationship. Moreover, comparing seasonality peaks does not necessarily reveal if the transmission is high, because high transmission may be associated with a relatively lower seasonality peak, but the transmission might be prolonged. So we used the average age at infection, which was not affected by the reporting fraction, to study the variation of HFMD transmission in China. The second reason for conflicting results could be the high correlation among the factors. Collinearity would prevent the identification of determining factors. The third reason for conflicting results might be the existence of an interaction between the population density and the public health performance. The population density effects on the average age at infection varied for different public health performance levels.

In this paper, we studied the effects of three classes of factors on the spatial pattern of HFMD transmission. We did not examine whether these significant factors also drove the incidence seasonality. The identification of driving factors of the annual seasonality HFMD will be the objective of our next study. Because we did not know exactly how much public health intervention reduced the level of the three types of factors, this study did not assess the effectiveness of the different control measures adopted in the country. We only identified the factors and interactions that significantly affected the average age at infection.

## Supporting Information

S1 File Division of 31 provinces in China into two groups. (DOCX)

S2 File Pearson’s correlation coefficients for each factor with the average age at infection for the entire country and the southeastern region. (DOCX)

S3 File Tables for correlation coefficients of factors. (DOCX)

S4 File VIF values of each step of the model. (DOCX)

## References

[1] Ma E, Lam T, Chan KC, Wong C, Chuang SK. Changing Epidemiology of hand, foot, and mouth disease in Hong Kong, 2001–2009. Japanese Journal of Infectious Disease 2010; 63: 422–426. 21099093

[2] Hosoya M, Kawasaki Y, Sato M, Honzumi K, Hayashi A, Hiroshima T, et al Genetic deversity of enterovirus 71 associated with hand, foot and mouth disease epidemics in Japan from 1983 to 2003. The Pediatric Infectious Disease Journal 2006; 25: 691–694. 10.1097/01.inf.0000227959.89339.c3 16874167

[3] Xing W, Liao Q, Viboud C, Zhang J, Sun J, Wu JT, et al Hand, foot, and mouth disease in China, 2008–12: an epidemiological study. The Lancet Infectious Diseases 2014; 14: 308–318. 10.1016/S1473-3099(13)70342-6 24485991 PMC4035015

[4] Wang Y, Feng Z, Yang Y, Self S, Gao Y, Longini IM, et al Hand, foot and mouth disease in China: patterns of spread and transmissibility during 2008–2009. Epidemiology 2011; 22: 781–792. 10.1097/EDE.0b013e318231d67a 21968769 PMC3246273

[5] Wang JF, Guo Y, Christakos G, Yang W, Liao Y, Li Z, et al Hand foot and mouth disease: spatiotemporal transmission and climate. International Journal of Health Geographics 2011; 10: 25 10.1186/1476-072X-10-25 21466689 PMC3079592

[6] Bo YC, Song C, Wang J, Li X. Using an autologistic regression model to identify spatial risk factors and spatial risk patterns of hand, foot and mouth disease (HFMD) in Mainland China. BMC Public Health 2014; 14: 358 10.1186/1471-2458-14-358 24731248 PMC4022446

[7] Wang J, Cao Z, Zeng DD, Wang Q, Wang X, Qian H. Epidemiological analysis, detection, and comparison of space-time patterns of Beijing hand-foot-mouth disease (2008–2012). PLoS ONE 2014; 9:e92745 10.1371/journal.pone.0092745 24663329 PMC3963949

[8] Liu Y, Wang X, Liu Y, Sun D, Ding S, Zhang B, et al Detecting Spatial-Temporal Clusters of HFMD from 2007 to 2011 in Shandong Province, China. PLoS ONE 2013; 8(5): e63447 10.1371/journal.pone.0063447 23704909 PMC3660332

[9] Liu W, Ji H, Shan J, Bao J, Sun Y, Li J, et al Spatiotemporal dynamics of hand-foot-mouth Disease and its relationship with meteorological factors in Jiangsu province, China. PLoS ONE 2015; 10: e0131311 10.1371/journal.pone.0131311 26121573 PMC4488144

[10] Huang Y, Deng T, Yu S, Gu J, Huang C, Xiao G, et al Effect of meteorological variables on the incidence of hand, foot and mouth disease in children: a time-series analysis in Guangzhou, China. BMC Infect Dis. 2013; 13:134 10.1186/1471-2334-13-134 23497074 PMC3626782

[11] Hu M, Li Z, Wang J, Jia L, Liao Y, Lai S, et al Determinants of the Incidence of Hand, Foot and Mouth Disease in China Using Geographically Weighted Regression Models. PLoS ONE 2012; 7: e38978 10.1371/journal.pone.0038978 22723913 PMC3377651

[12] Huang J, Wang J, Bo Y, Xu C, Hu M, Huang D. Identification of health risks of hand, foot and mouth disease in China using the geographical detector technique. International Journal of Environmental Research and Public Health 2014; 11: 3407–3423. 10.3390/ijerph110303407 24662999 PMC3987041

[13] Sabanathan S, Tan le V, Thwaites L, Wills B, Qui PT, Rogier van DH. Enterovirus 71 related severe hand, foot and mouth disease outbreaks in south-east Asia: current situation and ongoing challenges. Journal of Epidemiology and Community Health 2014; 68: 500–502. 10.1136/jech-2014-203836 24652348 PMC4033151

[14] Zhao J, Jiang F, Zhong L, Sun J, Ding J. Age patterns and transmission characteristics of Hand, Foot and Mouth disease in China. BMC Infectious Diseases, in press. 10.1186/s12879-016-2008-y PMC5117511 27871252

[15] Williams JR, Manfredi P, Butler AR, Ciofi degli Atti M, Salmaso S. Heterogeneity in regional notification patterns and its impact on aggregate national case notification data: the example of measles in Italy. BMC Public Health 2003; 3:23 10.1186/1471-2458-3-23 12871599 PMC194854

[16] Metcalf CJE, Bjornstad ON, Ferrari MJ, Klepac P, Bharti N, Lopez-Gatell H, et al The epidemiology of rubella in Mexico: seasonality, stochasticity and regional variation. Epidemiology and Infection 2010; 139: 1029–1038. 10.1017/S0950268810002165 20843389 PMC3884048

[17] Vynnycky E, White RD. An introduction to infectious disease modelling Oxford and New York: Oxford University Press; 2010.

[18] Anderson RM, May RM. Infectious diseases of humans: dynamics and control Oxford and New York: Oxford University Press; 1991.

[19] Rohani P, Zhong X, King AA. Contact Network Structure Explains the Changing Epidemiology of Pertussis. Science 2010; 330: 982–985. 10.1126/science.1194134 21071671

[20] McLean AR, Anderson RM. Measles in developing -countries I. The predicted impact of mass vaccination. Epidemiology and Infection 1988; 100: 111–133. 3378585 10.1017/s0950268800067170 PMC2249353

[21] McLean AR, Anderson RM. Measles in developing -countries II. The predicted impact of mass vaccination. Epidemiology and Infection 1988; 100: 419–442. 10.1017/s0950268800067170 3378585 PMC2249353

[22] Ferrari MJ, Grenfell BT, Strebel PM. Think globally, act locally: the role of local demographics and vaccination coverage in the dynamic response of measles infection to control. Philosophical Transactions of Royal Society B 2012; 368: 20120141 10.1098/rstb.2012.0141 23798689 PMC3720039

[23] Manfredi P, Cleur EM, Williams JR, Salmaso S, degli Atti MC. The pre-vaccination regional epidemiological landscape of measles in Italy: contact patterns, effort needed for eradication, and comparison with other regions of Europe. Population Health Metrics 2005; 3: 1 10.1186/1478-7954-3-1 15717921 PMC554971

[24] Li S, Eisenberg JNS, Spicknall IH, Koopman JS. Dynamics and Control of Infections Transmitted From Person to Person Through the Environment. American Journal of Epidemiology 2009; 170: 257–265. 10.1093/aje/kwp116 19474071

[25] Zhao J, Eisenberg J, Spicknall IH, Li S, Koopman JS. Model analysis of fomite mediated influenza transmission. PLoS ONE 2012; 7: e51984 10.1371/journal.pone.0051984 23300585 PMC3531458

[26] Grassly NC, Fraser C, Wenger J, Deshpande JM, Sutter RW, Heymann DL, et al New Strategies for the elimination of Polio from India. Science 2006; 314: 1150–1153. 10.1126/science.1130388 17110580

[27] Webster P. A polio-free world? Lancet 2005; 366: 359–320. 10.1016/s0140-6736(05)67008-0 16060049

[28] Ferrari MJ, Grais RF, Bharti N, Conlan AJK, Bjornstad ON, Wolfson LJ, et al The dynamics of measles in sub-Saharan Africa. Nature 2008; 451: 679–684. 10.1038/nature06509 18256664

[29] The Data-center of China Public Health Science. Available: http://www.phsciencedata.cn/Share/en/index.jsp. Accessed 2014 Sep 1.

[30] China Meteorological Administration. Available: http://www.cma.gov.cn/en2014. Accessed 2015 Sep 1.

[31] National Bureau of Statistics of the People's Republic of China. Available: http://www.stats.gov.cn. Accessed 2015 Sep 1.

[32] Liu Y, Rao K, Wu J, Gakidou E. Health System Reform in China 7 China’s health system performance. Lancet 2008; 372: 1914–1923. 10.1016/S0140-6736(08)61362-8 18930536

[33] Martinez-Bakker M, King AA, Rohani P. Unraveling the transmission ecology of polio. PLoS Biology 13(6):e1002172 10.1371/journal.pbio.1002172 26090784 PMC4474890

[34] Ma E, Lam T, Wong C, Chuang SK. Is hand, foot and mouth disease associated with meteorological parameters? Epidemiology & Infection 2010; 138: 1779–1788. 10.1017/S0950268810002256 20875200

[35] Pica N, Bouvier NM. Environmental factors affecting the transmission of respiratory viruses. Current Opinion in Virology 2012; 2: 90–95. 10.1016/j.coviro.2011.12.003 22440971 PMC3311988

[36] Neter J, Wasserman W, Kutner MH. Applied linear regression models Homewood, IL: Irwin; 1989.

[37] Xie YH, Chongsuvivatwong V, Tan Y, Tang Z, Sornsrivichai V, McNeil EB. Important roles of public playgrounds in the transmission of hand, foot and mouth disease. Epidemiology and Infection 2015; 143: 1432–1441. 10.1017/S0950268814002301 25170900 PMC9507205

