# Supplementary material for: Determinants of the Transmission Variation of Hand, Foot and Mouth Disease in China
Source: PLoS One. 2016 Oct 4;11(10):e0163789. doi: 10.1371/journal.pone.0163789 (PMC5049751; doi:10.1371/journal.pone.0163789)
Supplement: S1 File — (DOCX) [file pone.0163789.s001.docx]

**S1 File. Division of 31 provinces in China into two groups.**

We divided 31 provinces of China into two groups: nine provinces in the northwestern region and 22 provinces in the southeastern region.

Provinces in the northwestern region include Heilongjiang, Jilin, Liaoning, Inner Mongolia, Ningxia, Gansu, Qinghai, Xinjiang, Tibet.

Provinces in the southeastern region include Beijing, Tianjin, Hebei, Shanxi, Shandong, Shaanxi, Henan, Jiangsu, Anhui, Shanghai, Hubei, Sichuan, Chongqing, Zhejiang, Hunan, Jiangxi, Guizhou, Yunnan, Fujian, Guangxi, Guangdong, Hainan.
