# Supplementary material for: Determinants of the Transmission Variation of Hand, Foot and Mouth Disease in China
Source: PLoS One. 2016 Oct 4;11(10):e0163789. doi: 10.1371/journal.pone.0163789 (PMC5049751; doi:10.1371/journal.pone.0163789)
Supplement: S2 File — (DOCX) [file pone.0163789.s002.docx]

**S2 File. Pearson’s correlation coefficients for each factor with the average age at infection for the entire country and the southeastern region.**

Table A. Pearson’s correlation coefficient between each factor and the average age at infection

|  | Country | Southeastern region |
| --- | --- | --- |
| Factors | Coefficient (*p*-value) | Coefficient (*p*-value) |
| Mean temperature | -0.8053 (0.0000)* | -0.5411 (0.0093)* |
| Relative humidity | -0.7409 (0.0000)* | -0.5732 (0.0053)* |
| Rainfall | -0.7744 (0.0000)* | -0.6067 (0.0028)* |
| Sunshine | -0.7571 (0.0000)* | -0.6080 (0.0027)* |
| Log (population density) | -0.5811 (0.0006)* | 0.4853 (0.0221)* |
| Health System Performance | -0.0948 (0.6118) | 0.3134 (0.1555) |
| Birth rate | -0.1908 (0.3038) | -0.7461 (0.0000)* |
| Per capita GRP | 0.1179 (0.5276) | 0.5949 (0.0035)* |
