# Supplementary material for: Determinants of the Transmission Variation of Hand, Foot and Mouth Disease in China
Source: PLoS One. 2016 Oct 4;11(10):e0163789. doi: 10.1371/journal.pone.0163789 (PMC5049751; doi:10.1371/journal.pone.0163789)
Supplement: S3 File — (DOCX) [file pone.0163789.s003.docx]

**S3 File. Tables for correlation coefficients of factors.**

Table B. Correlation coefficients of factors in 30 provinces

|  | Temp | RH | Rain | Sun | Log PD | Birth | Health | GRP |
| --- | --- | --- | --- | --- | --- | --- | --- | --- |
| Temp | 1 | 0.78163 | 0.804268 | 0.865688 | 0.539451 | 0.285301 | 0.275579 | 0.019555 |
| RH | 0.7816 | 1.0000 | 0.9953 | 0.8673 | 0.3700 | 0.2234 | 0.0719 | -0.2119 |
| Rain | 0.8043 | 0.9953 | 1.0000 | 0.8818 | 0.4037 | 0.2330 | 0.0810 | -0.2193 |
| Sun | 0.8657 | 0.8673 | 0.8818 | 1.0000 | 0.4023 | 0.2318 | 0.2230 | -0.0531 |
| LogPD | 0.5395 | 0.3700 | 0.4037 | 0.4023 | 1.0000 | -0.3803 | 0.6344 | 0.5664 |
| Birth | 0.2853 | 0.2234 | 0.2330 | 0.2318 | -0.3803 | 1.0000 | -0.5473 | -0.5560 |
| Health | 0.2756 | 0.0719 | 0.0810 | 0.2230 | 0.6344 | -0.5473 | 1.0000 | 0.6550 |
| GRP | 0.0196 | -0.2119 | -0.2193 | -0.0531 | 0.5664 | -0.5560 | 0.6550 | 1.0000 |

Table C. Correlation coefficients of factors in 22 provinces in the southeastern region

|  | Temp | RH | Rain | Sun | Log PD | Birth | Health | GRP |
| --- | --- | --- | --- | --- | --- | --- | --- | --- |
| Temp | 1 | 0.711132 | 0.720864 | 0.843908 | -0.13637 | 0.516216 | 0.185205 | -0.17195 |
| RH | 0.7111 | 1.0000 | 0.9958 | 0.8157 | -0.3070 | 0.5144 | -0.1556 | -0.3995 |
| Rain | 0.7209 | 0.9958 | 1.0000 | 0.8338 | -0.3180 | 0.5524 | -0.1786 | -0.4226 |
| Sun | 0.8439 | 0.8157 | 0.8338 | 1.0000 | -0.1459 | 0.5127 | 0.0827 | -0.2042 |
| LogPD | -0.1364 | -0.3070 | -0.3180 | -0.1459 | 1.0000 | -0.5957 | 0.5830 | 0.8855 |
| Birth | 0.5162 | 0.5144 | 0.5524 | 0.5127 | -0.5957 | 1.0000 | -0.4315 | -0.7227 |
| Health | 0.1852 | -0.1556 | -0.1786 | 0.0827 | 0.5830 | -0.4315 | 1.0000 | 0.7339 |
| GRP | -0.1720 | -0.3995 | -0.4226 | -0.2042 | 0.8855 | -0.7227 | 0.7339 | 1.0000 |

Table D. Correlation coefficients of factors in eight provinces in the northwestern region

|  | Temp | RH | Rain | Sun | Log PD | Birth | Health | GRP |
| --- | --- | --- | --- | --- | --- | --- | --- | --- |
| Temp | 1.0000 | -0.2317 | -0.1479 | -0.3974 | 0.4889 | 0.0956 | 0.2773 | 0.2116 |
| RH | -0.2317 | 1.0000 | 0.9772 | 0.5902 | 0.3864 | -0.3509 | 0.2141 | -0.2684 |
| Rain | -0.1479 | 0.9772 | 1.0000 | 0.6071 | 0.5055 | -0.3838 | 0.3074 | -0.3324 |
| Sun | -0.3974 | 0.5902 | 0.6071 | 1.0000 | 0.4351 | -0.8146 | 0.6117 | 0.2681 |
| LogPD | 0.4889 | 0.3864 | 0.5055 | 0.4351 | 1.0000 | -0.6474 | 0.8911 | 0.2116 |
| Birth | 0.0956 | -0.3509 | -0.3838 | -0.8146 | -0.6474 | 1.0000 | -0.7815 | -0.5618 |
| Health | 0.2773 | 0.2141 | 0.3074 | 0.6117 | 0.8911 | -0.7815 | 1.0000 | 0.4772 |
| GRP | 0.2116 | -0.2684 | -0.3324 | 0.2681 | 0.2116 | -0.5618 | 0.4772 | 1.0000 |
