# Supplementary material for: Determinants of the Transmission Variation of Hand, Foot and Mouth Disease in China
Source: PLoS One. 2016 Oct 4;11(10):e0163789. doi: 10.1371/journal.pone.0163789 (PMC5049751; doi:10.1371/journal.pone.0163789)
Supplement: S4 File — (DOCX) [file pone.0163789.s004.docx]

**S4 File. VIF values of each step of the mode**l. We first used the full set of factors plus the interaction factors of the log of the population density, the Health System Performance and the per capita GRP. The VIF value for each factor was calculated, and the one with the highest value was removed. A backward approach was used until all VIF values were below the threshold of 5.

Table E. VIF values calculated in each step of the model for the entire country

| Factor | Rain | GRP per capita | Tempe-rature | Sun | Relative  humidity | Log of  population density | Health  System  Performance | Birth | PD:H | H:G | PG:H:G |
| --- | --- | --- | --- | --- | --- | --- | --- | --- | --- | --- | --- |
| VIF | 370.4 | 14.2 | 9.4 | 10.0 | 285.7 | 7.4 | 3.2 | 5.4 | 7.3 | 2.2 | 1.2 |
| VIF (Rain removed) | | 14.0 | 9.4 | 7.3 | 7.4 | 5.4 | 3.0 | 5.1 | 6.6 | 1.9 | 1.1 |
| VIF (GDP removed) | | | 8.8 | 7.0 | 5.3 | 3.7 | 2.6 | 3.2 | 1.3 | 1.3 | 1.1 |
| VIF (Temperature removed) | | | | 5.5 | 5.0 | 2.4 | 2.1 | 1.8 | 1.2 | 1.3 | 1.0 |
| VIF (Sun removed) | | | | | 1.6 | 2.4 | 2.1 | 1.8 | 1.2 | 1.3 | 1.0 |

PD:H is the interaction of the log of the population density and the Health System Performance;

H:G is the interaction of the Health System Performance and the per capita GRP;

PD:H:G is the interaction of the log of the population density, the Health System Performance and the per capita GRP;

Table F. VIF values calculated in each step of the model for the southeastern region

| Factor | Rain | GRP per capita | Sun | Tempe-rature | Relative  humidity | Log of  population density | Health  System  Performance | Birth | PD:H | H:G | PG:H:G |
| --- | --- | --- | --- | --- | --- | --- | --- | --- | --- | --- | --- |
| VIF | 228.7 | 23.6 | 7.3 | 4.7 | 194.5 | 10.1 | 4.1 | 3.8 | 4.4 | 3.0 | 1.1 |
| VIF (Rain removed) | | 22.7 | 5.7 | 4.7 | 4.2 | 9.4 | 4.1 | 3.6 | 4.4 | 3.0 | 1.1 |
| VIF (GDP removed) | | | 5.7 | 4.6 | 3.8 | 2.0 | 2.2 | 2.8 | 2.1 | 2.1 | 1.1 |
| VIF (Sun removed) | | | | 3.5 | 2.6 | 2.0 | 2.2 | 2.7 | 2.1 | 2.0 | 1.1 |

PD:H is the interaction of the log of the population density and the Health System Performance;

PD:G is the interaction of the log of the population density and the per capita GRP;

PD:H:G is the interaction of the log of the population density, the Health System Performance and the per capita GRP;
